# Comparison of Advanced Threshold and SITA Fast Perimetric Strategies

**DOI:** 10.1155/2020/7139649

**Published:** 2020-12-23

**Authors:** Bartosz L. Sikorski, Adriana Laudencka

**Affiliations:** ^1^Department of Ophthalmology, Nicolaus Copernicus University, 9 M. Sklodowskiej-Curie St., Bydgoszcz 85-309, Poland; ^2^Oculomedica Eye Research & Development Center, 9 Broniewskiego St., Bydgoszcz 85-090, Poland

## Abstract

**Purpose:**

To compare the results obtained with two threshold strategies of visual field assessment: Humphrey SITA Fast (SFA) (Carl Zeiss Meditec) and PTS 2000 Advanced Threshold (ADV) (Optopol Technology) in healthy subjects and patients with glaucoma.

**Methods:**

The study sample comprised of 53 healthy volunteers and 69 patients with glaucoma. One eye of each patient was examined with the SFA and ADV strategies. The quantitative comparisons of test duration and global indices were made using correlation coefficients. The sensitivity and specificity of the algorithms were evaluated based on the GHT results and the adjusted Anderson–Patella (A&P) criteria.

**Results:**

The ADV test duration was shorter both in healthy subjects (by 5%) and patients with glaucoma (by 18%). The mean differences in MS values between the SFA and the ADV strategies were 1.06 ± 1.13 dB (MS_SFA_-MS_ADV_) in healthy subjects and 1.00 ± 1.92 dB (MS_SFA_-MS_ADV_) in patients with glaucoma. The MD index of ADV tests was lower than the SFA in the healthy (−0.74 ± 1.09 dB) (MS_SFA_-MS_ADV_) and glaucoma group (−0.85 ± 2.19 dB) (MS_SFA_-MS_ADV_). The mean differences in PSD values determined using both methods were −0.86 ± 0.67 dB (PSD_SFA_-PSD_ADV_) and −0.53 ± 1.48 dB (PSD_SFA_-PSD_ADV_) in healthy subjects and patients with glaucoma, respectively. Analysis of receiver operating characteristic curves built from MD and PSD indices show bigger area under curve in SFA than in ADV (0.983 vs.0.968 and 0.986 vs. 0.938, respectively). The GHT-based sensitivity and specificity for the ADV strategy were 92.75% and 77.36%, respectively, as compared to 92.75% and 90.57%, respectively, for the SFA strategy.

**Conclusions:**

Both SFA and ADV enable effective identification of glaucomatous defects within 5 minutes. The ADV strategy, however, is significantly faster. The correlation between the global indices of SFA and ADV is very high. Both strategies offer very high sensitivity when using both GHT and A&P criteria.

In the age of rapid development of OCT-based diagnostic instruments in ophthalmology, the automated perimetry is still the fundamental method of visual field assessment. It provides not only an estimation of retinal sensitivity but can also confirm the effect of all pathological findings demonstrated using other diagnostic tests on the patient's functional vision. However, perimetry is a subjective method, and as such, it is totally dependent on patient cooperation. Subjects with poor reliability of responses will produce unreliable results. It was proven that patient fatigue during the test is one of the key factors, which affect the reliability of its result. One of the main goals of researchers who worked on automation of perimetry back in the 1970s was to optimize the threshold evaluation strategy [1–3]. The results of their work laid a foundation for subsequent research and, with some further optimization, have been implemented in modern perimetry [4–7]. The basic approach called “bracketing” alters stimulus intensity in a step-wise manner, until the threshold is crossed and the threshold estimation error is acceptable. This approach produces an accurate result but at the expense of long test duration which adversely affects patient concentration and can ultimately impair result reliability [8–10]. Since the development of the first automated perimeter over 40 years ago, there have been several breakthrough moments which made perimetry faster and more reliable. One of such moments was the introduction of the Dynamic strategy in Octopus perimeters (Interzeag AG, Schlieren, Switzerland). The Dynamic strategy has reduced the examination time by 30–50% compared to standard thresholding [11, 12]. Another revolutionary approach was the Tendency-Oriented Perimetry (TOP), which shortened assessment duration by testing each field point once [13]. The TOP approach is additionally associated with only minimum fatigue effect and still offers good sensitivity as compared to the standard strategy [10, 14, 15]. One of the “new-generation strategies” which have gained great popularity is the Swedish Interactive Thresholding Algorithm (SITA) [16]. Initially, two SITA variants were developed in order to significantly shorten assessment duration without affecting data quality: SITA Standard and SITA Fast (SFA). After two decades of SITA use with the Humphrey Field Analyzer (HFA, Carl Zeiss Meditec AG, Germany), the new variant of SITA named SITA Faster (SFR) was introduced. It is said to reduce the test duration even further than SFA [17]. The manufacturers of modern perimeters are still working towards developing the perfect strategy that yields a reliable test result within the shortest possible timeframe. The examples of their attempt include strategies such as GATE, ZATA, and SPARK [18–20]. As a part of the same quest, the family of PTS perimeters (Optopol Technology, Poland) operates based on a proprietary testing strategy known as Advanced Threshold (ADV), which aims at reducing examination time to that of screening strategies, without sacrificing result quality.

The aim of the paper is to compare the results obtained with HFA SFA and PTS ADV strategies in healthy volunteers and subjects with glaucoma. The sensitivity and specificity of both tests in detecting visual defects in patients with glaucoma were also assessed.

## 1. Materials and Methods

### 1.1. Subjects and the Study Protocol

The study group comprised of 135 healthy volunteers and glaucoma patients recruited from ophthalmic clinic. None had diabetes, cataract, and corneal or retinal disease which could affect test results. Patients were classified as healthy when they had (1) no suspicious disc changes, (2) no family history of glaucoma, (3) normal intraocular pressure, (4) refractive error below 5 dioptres sphere and 3 dioptres cylinder, and (5) best corrected visual acuity of 0.5 or better. All participants additionally had visual field performed twice within 2 weeks prior to study commencement. The glaucoma group was selected based on the following criteria: (1) patients with glaucomatous disc changes and intraocular pressure >21 mmHg, (2) refractive error below 5 dioptres sphere and 3 dioptres cylinder, (3) best corrected visual acuity of 0.5 or better, and (4) previous experience of automated perimetry. One eye of each subject was enrolled in the study. If both eyes were eligible, a random choice was made. Each patient underwent a series of two tests on HFA II 750i and Optopol PTS 2000 perimeters. Both devices utilized the gold standard testing parameters: Goldman stimulus of size III, white colour, maximum intensity of 10000 asb, 300 mm aspherical bowl, and background illumination of 31.5 asb. The default duration of the stimulus exposure was 200 ms in HFA II 750i and 250 ms in PTS 2000. The HFA device used the SFA strategy whereas the PTS 2000 used the ADV strategy. All tests were performed on 24-2 test field, without fovea and short-fluctuation (SF) testing. There was a 10- minute break between the tests within the series, and the order of the tests within the series was randomized to avoid the fatigue effect bias. The appropriate spectacle correction was used for all tests. The reliability of the tests was evaluated with use of false-positive (FP), false-negative (FN), and blind-spot monitoring. The HFA SFA results were considered unreliable if any of FP, FN, and fixation loses (FL) error indexes exceeded 25%. The PTS ADV results were considered unreliable, if more than one error index exceeded 25%. The results were included in the analysis only if both test results for a given eye were reliable. Thus, 122 eyes were selected for further assessment. The healthy group contained 53 healthy eyes (30 left and 23 right, 17 men and 36 women; age 30–84 years, mean age 52.5 ± 13.0), whereas the glaucoma group consisted of 69 glaucomatous eyes (36 left and 33 right, 25 men and 44 women; age 40–89 years, mean age 68.7 ± 12.7). The mean age of glaucoma patients was significantly higher than that of the healthy subjects (*p* < 0.0001).

Each examination result was imported to the statistical analysis software (Statistica 13.1, Dell Inc. USA) maintaining information about raw sensitivity value at all test locations except 2 points within the blind spot area. The following information was extracted: mean test duration, mean sensitivity (MS) index, mean difference between the SFA MS and ADV MS, root mean square (RMS) of differences between threshold estimate in the SFA and ADV strategies, mean defect (MD) index, mean difference between the SFA MD and ADV MD, pattern standard deviation (PSD) index, mean difference between SFA PSD and ADV PSD, visual field index (VFI)/visual quality index (VQi), mean difference between VFI and VQi, and the Glaucoma Hemifield Test (GHT) result. The correlation between the SFA and ADV global indices was then analysed. Each result was also assigned the Anderson–Patella (A&P) classification index (0/2, 1/2, 2/2) depending on how many criteria it met [21]. The first criterion is the GHT index “outside normal limits.” The second one is the cluster of 3 or more points with probability value <5% with, at least, one of them being <1% (excluding the 30-2 field edge points and points surrounding the blind spot point). The third criterion is the PSD value outside the normal 95% of population. This criterion, however, can only be used with SFA results, due to the lack of statistical data for the PSD index in ADV tests. Based on the number of the A&P criteria they met, the results were classified as 0/2, 1/2 and 2/2. The specificity and sensitivity testing was performed two-fold. The first assumption was to consider only the results from A&P group 2/2 as glaucomatous, whereas the second one included both 1/2 and 2/2 results considering them as glaucomatous. Similarly, the sensitivity and specificity testing of the stand-alone GHT index was performed two-fold. In the first mode, all GHT outside normal limits were considered glaucomatous. In the second one, all GHT outside normal limits and/or borderline were considered glaucomatous. Finally, the RMS error in threshold estimates between the SFA and ADV strategies was evaluated for each tested location. The diagnostic performance of the SFA and ADV was assessed by using receiver operating characteristic (ROC) curves and the area under the ROC curve analysis. To assess agreement between the SFA and ADV for MS, MD, and PSD, the Bland–Altman plots were performed and 95% limits of agreement (LoA) were calculated by the mean difference ± 1.96SD [22]. The study protocol was in accordance with the Declaration of Helsinki, and the Institutional Ethics Committee approval was obtained.

### 1.2. The Visual Field Testing Algorithms

#### 1.2.1. HFA SITA Fast (SFA)

The SITA algorithm is based on the Bayesian posterior probability distribution functions. In its core, SITA uses both staircase and maximum-likelihood threshold procedures. For each test, there are two Bayesian posterior probability distributions calculated and constantly updated throughout the test, that is, normal and glaucomatous. The initial models are built based on the prior knowledge of age-corrected normal thresholds and random intersubject visual field variability [23, 24]. The peak of the distribution represents the threshold value with maximum posterior probability. The width of the distribution is a basis for evaluating the accuracy of the current threshold estimate. The shape of posterior distribution of threshold values changes based on patient responses to stimuli. After each response, the distributions at the tested locations are updated based on frequency-of-seeing curves. Additionally, probability distributions at adjacent locations are updated based on correlations between thresholds at different test point locations. The maximum posterior estimates, best threshold, and their accuracy are available during the test for each location and can be utilized to stop the algorithm when error level is accepted. In the SITA Standard variant of the algorithm, the actual intensities of exposed stimuli are altered in a classical 4-2 staircase procedure. Before the thresholding is terminated, there needs to be, at least, one reversal in intensity alteration. The algorithm ends when the estimation error is smaller than the defined acceptance level or after two reversals in intensity alteration have occurred. In the SFA variant of the algorithm, the acceptance level of the threshold estimation error was increased compared to the SITA standard. This reduced the test duration even further at the expense of its accuracy. In both variants of SITA, the threshold displayed as a result is the most likely mode of the two probability distributions. Therefore, the threshold value displayed by SITA may be higher than the actual intensity noticed by a patient, unlike in the full threshold strategy, where the displayed value is the last seen intensity [8]. One additional factor which affects test duration in SITA is the absence of traditional FP tests, which are replaced by recording patient responses with extremely short reaction time (<200 ms) and combining them with probability methods to obtain the % FP value [25]. The latest SITA variant (the SFR strategy) is said to reduce the test duration by about 30% compared to the SFA [17]. This is achieved thanks to several modifications: adjusting the starting intensities at root points to age-corrected values, reducing the number of necessary threshold crossings in root points, updating the statistical models to better match the SITA characteristics, as well as resigning from double checking of blind points, the negative and bind spot catch trials, and adding extra pause time after nonseen stimuli.

#### 1.2.2. PTS Advanced (ADV)

The ADV algorithm is divided into 4 consecutive virtual stages [26]. The points examined at each stage cover the tested area evenly. The initial values of intensity levels in all locations are set according to the age-normative dataset. After each stimulus exposure, more information about visual field is acquired, which, by means of interpolation techniques, is utilized to update the actual intensity levels at points adjacent to the tested one. During the first stage, 25% of all test points are tested with a modified full threshold approach: 6–3 dB bracketing with a single threshold level crossing. If a patient sees the initial exposure, which is close to the age norm, then the intensity is decreased by 3 dB for the next exposure in this location. If the patient does not respond to the stimulus darker by 3 dB at the next exposure, then the value from initial exposure is considered a final result. Otherwise, the intensity is decreased by another 3 dB. The intensity is reduced by 3 dB in a step-wise manner until the sensitivity threshold is crossed. This way, for healthy locations, only 2 exposures are needed to estimate the result. In the second scenario, that is, when a patient does not respond to the initial exposure during the first stage of the ADV strategy, the stimulus intensity is increased by 6 dB in this location and a regular 6–3 dB bracketing is started to evaluate the sensitivity threshold. During the subsequent stages (second, third, and fourth), the remaining points are examined using a modified PTS screening algorithm. The initial values at each stage are calculated from the finished points. If a patient sees the stimulus exposed at the initial level, this value is accepted as a final threshold value and the point is not tested anymore. If a patient does not respond to the first exposure, the algorithm finds the threshold using the 6–3 dB bracketing with a single threshold crossing. After each stage, the results are utilized to update the adjacent points which are to be tested in next stage. Thus, completing all four stages reduces the odds of overlooking defects whilst preserving short test duration. The threshold displayed as a result at any stage of the ADV strategy is the highest intensity the patient responded to.

## 2. Results

The summary of visual field indices values (test duration, MS, MD, PSD, VFI, and VQi) is presented in Table 1.

### 2.1. Test Duration

**Figure 1:**
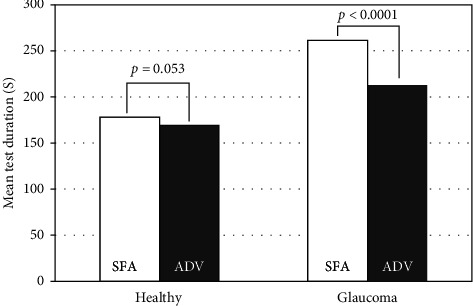
Mean SFA and ADV tests duration in the healthy and glaucoma group.

**Figure 2:**
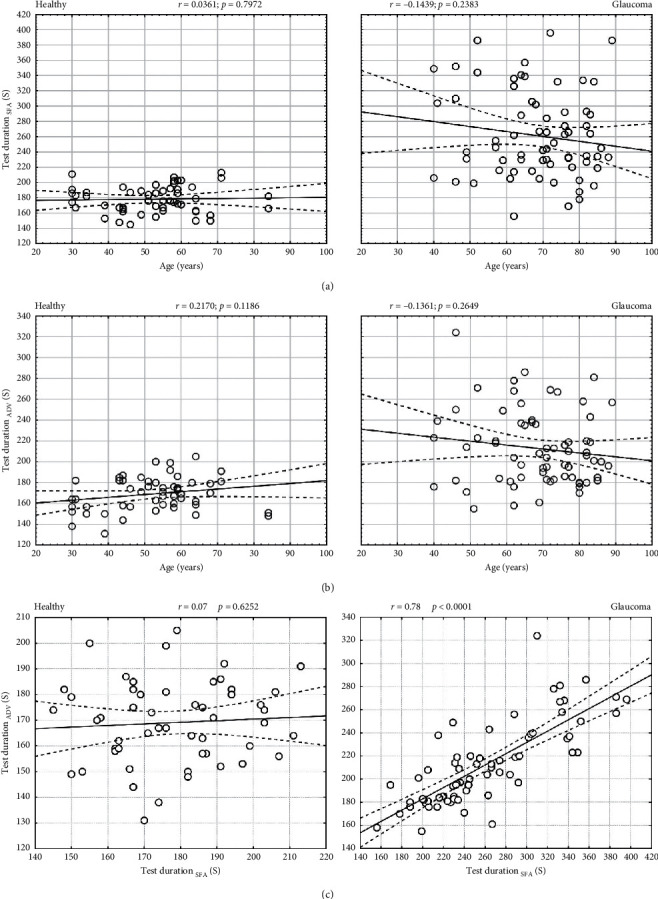
Test duration analysis in healthy and glaucomatous eyes. (a, b) Correlation of test duration with patients' age for SFA and ADV strategies; (c) correlation between test duration in ADV and SFA strategies.

The mean test duration with the HFA SFA strategy was 178 ± 17 s and 261 ± 57 s in healthy subjects and glaucoma patients, respectively. With the PTS ADV test, the mean time was 169 ± 16 s in healthy subjects and 213 ± 35 s in glaucoma patients (Figure 1). The mean test duration was significantly shorter with ADV than with SFA in both healthy subjects (*p* < 0.0053) and glaucoma patients (*p* < 0.0001). The mean test duration was significantly longer in glaucoma patients, both with SFA (*p* < 0.0001) and ADV (*p* < 0.0001). There was no correlation between patient age and the duration of SFA (*r* = 0.04, *p*=0.7972) or ADV (*r* = 0.22, *p*=0.1186) test in healthy subjects, as well as in glaucoma patients (SFA: *r* = -0.14, *p*=0.2383; ADV: *r* = −0.1361, *p*=0.2649) (Figures 2(a) and 2(b). There was a very strong correlation between test duration in ADV and SFA in the glaucoma group (*r* = 0.78, *p* < 0.0001) and no correlation in the healthy group (*r* = 0.07, *p*=0.6252) (Figure 2(c)).

### 2.2. Mean Sensitivity

**Figure 3:**
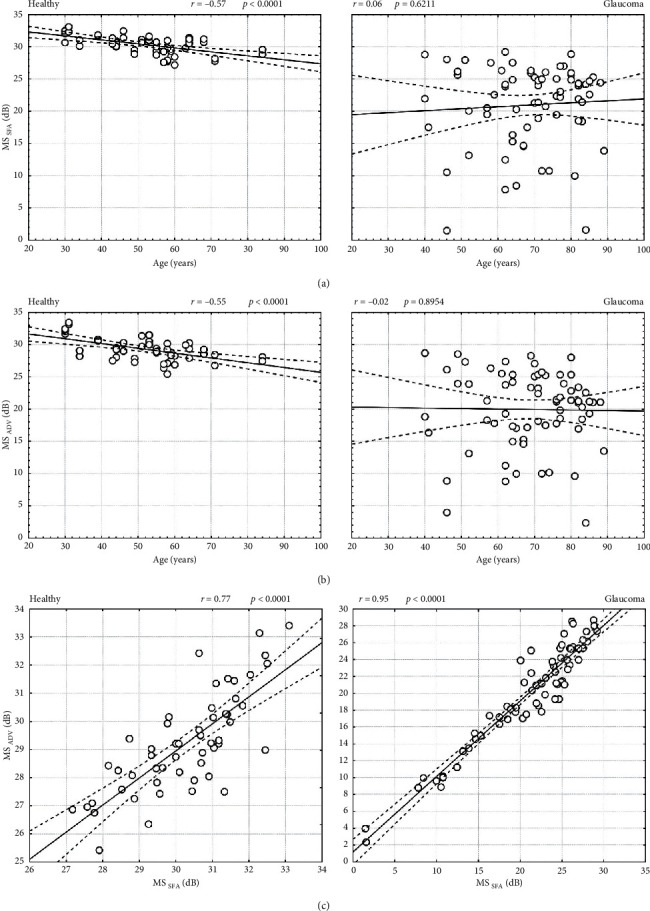
Mean sensitivity analysis in healthy and glaucomatous eyes. (a, b) Correlation of retinal MS with patient age for SFA and ADV strategies; (c) correlation between retinal MS in ADV and SFA strategies.

**Figure 4:**
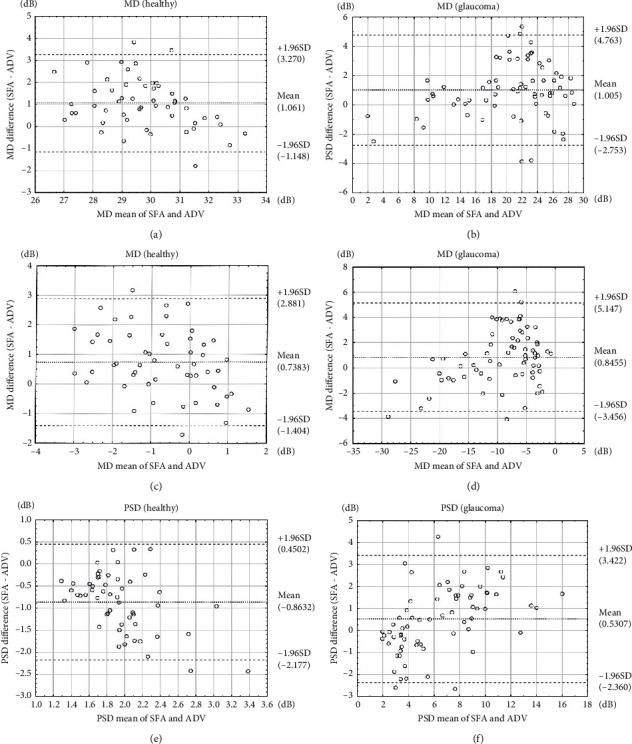
Bland–Altman plots for MS (a, b), MD (c, d), and PSD (e, f). The dashed lines indicate the 95% agreement interval.

The MS tested with SFA strategy was significantly higher than that obtained with ADV strategy both in healthy (*p* < 0.0001) and glaucoma group (*p* < 0.0001) (Table 1). The mean difference between MS values determined using SFA and ADV strategies (MS_SFA_-MS_ADV_) was 1.06 ± 1.13 dB and 1.00 ± 1.92 dB in the healthy and glaucoma group, respectively. This number did not differ significantly between the healthy and glaucoma group (*p*=0.8507). The MS index difference between SFA and ADV was systematic and independent of patient condition. There was a moderate negative correlation between patient age and MS in SFA (*r* = −0.57, *p* < 0.0001) and ADV (*r* = −0.55, *p* < 0.0001) in the healthy group, but no correlation between MS and patient age in SFA (*r* = 0.06, *p*=0.6211) and ADV (*r* = −0.02, *p*=0.8954) in the glaucoma group (Figures 3(a) and 3(b). There was a strong correlation between MS in ADV and SFA in the healthy group (*r* = 0.77, *p* < 0.0001, *S* = 0.91 dB) and a very strong such correlation in the glaucoma group (*r* = 0.95, *p* < 0.0001, *S* = 1.98 dB) (Figure 3(c)). The Bland–Altman analysis of the MS relation between SFA and ADV is presented in Figures 4(a) and 4(b). The mean MS measurement difference in healthy subjects was 1.061 dB (the 95% LoA ranged from −1.148 to 3.270), whereas for glaucoma patients, it was 1.005 dB (the 95% LoA ranged from −2.753 to 4.763).

### 2.3. RMS Deviation of Sensitivity Values

**Figure 5:**
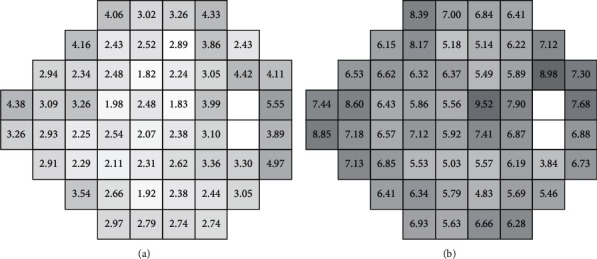
The mean RMS deviation at every test location in the healthy group (a) and the glaucoma group (b).

**Figure 6:**
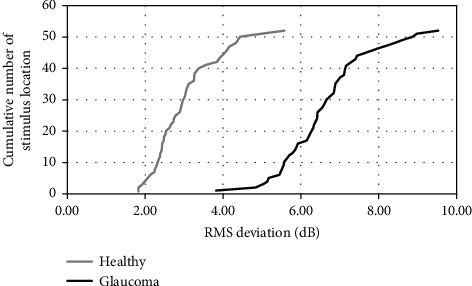
RMS deviation at each tested location cumulative plot in the healthy group and the glaucoma group.

The mean RMS of differences in sensitivity values between SFA and ADV (RMS_SFA_-RMS_ADV_) for all tested locations was 3.01 ± 0.84 dB in healthy subjects and 6.36 ± 2.07 dB in glaucomatous patients. The mean RMS was significantly higher in glaucomatous eyes than in healthy eyes (*p* < 0.0001). There was no correlation between patient age and RMS both in the healthy (*r* = 0.01, *p*=0.9394) and glaucoma group (r = −0.15, *p*=0.2037). The RMS value analysis with respect to test point locations in the healthy group showed that the highest error is at points adjacent to the blind spot area (Figure 5(a)). The increase of eccentricity yielded an increased RMS error. In the glaucoma group, there was no regularity in RMS changes (Figure 5(b)). There was one location with excessively high RMS value (coordinates *x* = 3, *y* = 3). Inspection of test results showed that this point was often at the sharp boundary between normal and depressed visual fields in advanced glaucoma cases and the estimated threshold fluctuated between the tests.  Figure 6 shows the cumulative plot of the RMS deviation at each location tested in the healthy and glaucoma groups.

### 2.4. Mean Deviation

**Figure 7:**
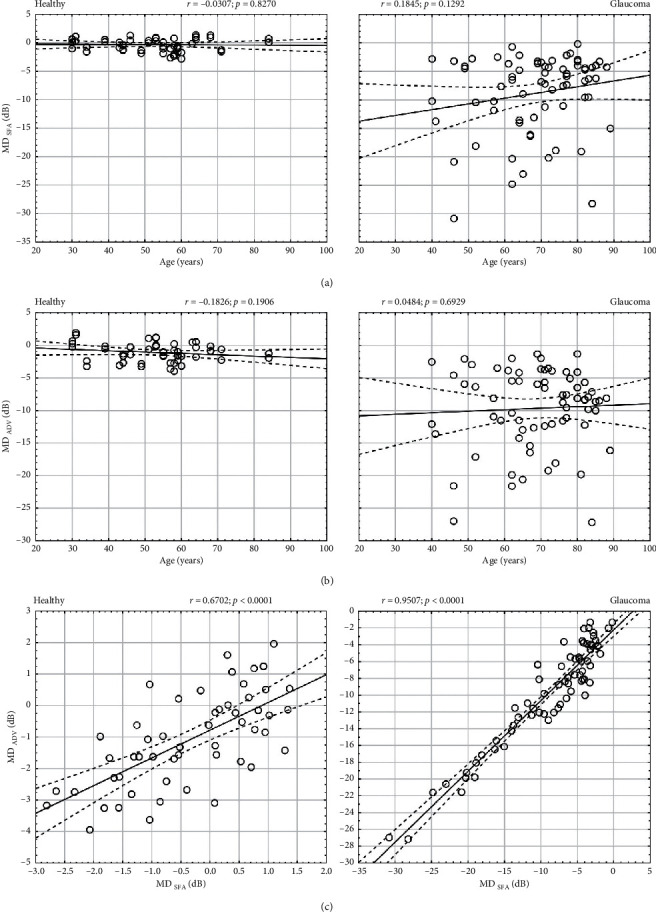
The analysis of mean deviation in healthy and glaucomatous eyes. (a, b) Correlation of SFA MD and ADV MD with patient age; (c) correlation between MD for ADV and MD for the SFA strategy.

The mean values of MD in SFA and in ADV differed in both the healthy (*p*=0.0021) and glaucomatous group (*p* < 0.0001) (Table 1). The difference between the subject sensitivity and age-normative data was bigger for ADV than for SFA. The MD values for ADV strategy were lower on average by −0.85 ± 2.19 dB (MD_SFA_-MD_ADV_) compared to SFA MD in the healthy group and by −0.74 ± 1.09 dB (MD_SFA_-MD_ADV_) in the glaucomatous group. The differences were not significant (*p*=0.7452). There was no correlation between patient age and MD in SFA (*r* = −0.03, *p*=0.8270) or MD in ADV (*r* = −0.18, *p*=0.1906) in the healthy or glaucoma group (SFA MD: *r* = 0.18, *p*=0.1292; ADV MD: *r* = 0.05, *p*=0.6929) (Figures 7(a) and 7(b). There was a very strong correlation between MD in ADV strategy and MD in SFA strategy in the glaucoma group (*r* = 0.95, *p* < 0.0001, *S* = 2.09 dB) and a strong correlation in the healthy group (*r* = 0.67, *p* < 0.0001, *S* = 0.84 dB) (Figure 7(c)). The Bland–Altman analysis of the MD relation between SFA and ADV is presented in Figures 4(c)–4(d)). The mean MD measurement difference in healthy subjects was 0.738 dB (the 95% LoA ranged from −1.404 to 2.881), whereas for glaucoma patients, it was 0.846 dB (the 95% LoA ranged from −3.456 to 5.147).

### 2.5. Pattern Standard Deviation

**Figure 8:**
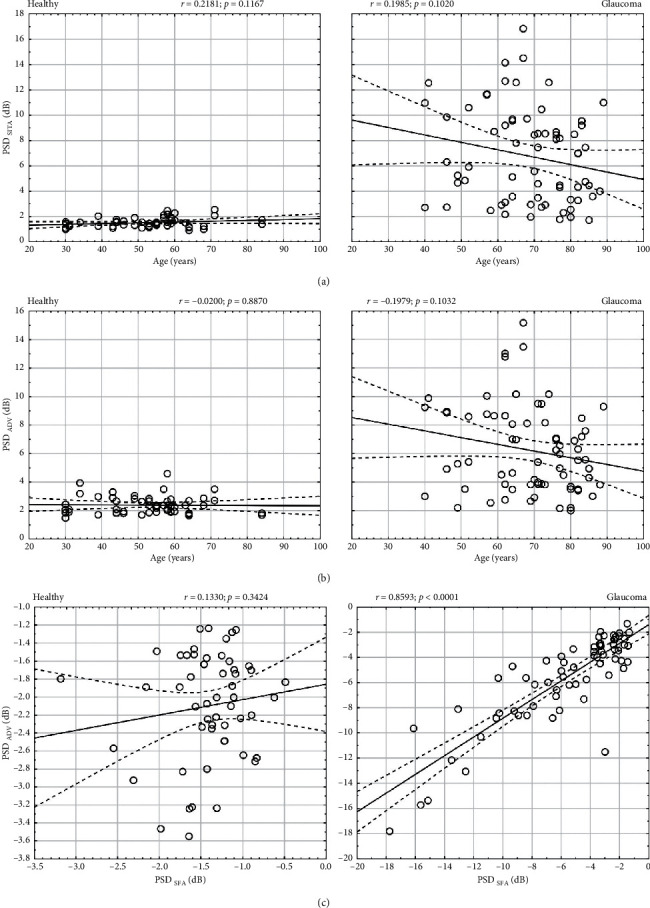
The analysis of pattern standard deviation in healthy and glaucomatous eyes. (a, b) Correlation of PSD with patient age for SFA and ADV strategies; (c) correlation between PSD in ADV and SFA strategies.

The mean values of PSD in SFA and ADV were significantly different in both the healthy (*p* < 0.0001) and glaucoma group (*p*=0.0039) (Table 1). The PSD values in ADV were higher than in SFA in healthy subjects with the difference of −0.86 ± 0.67 dB (PSD_SFA_-PSD_ADV_) and lower than in the SFA test in glaucoma patients with the difference of −0.53 ± 1.48 dB (PSD_SFA_-PSD_ADV_). The differences were significant (*p* < 0.0001). There was no correlation between patient age and PSD in SFA (*r* = 0.22, *p*=0.1167) and in ADV (*r* = −0.02, *p*=0.8870) in the healthy group, as well as between patient age and PSD in SFA (*r* = −0.20, *p*=0.1020) and in ADV (*r* = −0.20, *p*=0.1032) in the glaucoma group (Figures 8(a) and 8(b)). There was a very strong correlation between ADV PSD and SFA PSD in the glaucoma group (*r* = 0.86, *p* < 0.0001, *S* = 1.48 dB) but no such correlation in the healthy group (*r* = 0.13, *p*=0.3424, *S* = 0.37 dB) (Figure 8(c)). The Bland–Altman analysis of the PSD relation between SFA and ADV is presented in Figures 4(e) and 4(f). The mean PSD measurement difference in healthy subjects was −0.863 dB (the 95% LoA ranged from −2.177 to 0.450), whereas for glaucoma patients, it was 0.531 dB (the 95% LoA ranged from −2.360 to 3.422).

### 2.6. Receiver Operating Characteristic (ROC) Analysis of MD and PSD

**Figure 9:**
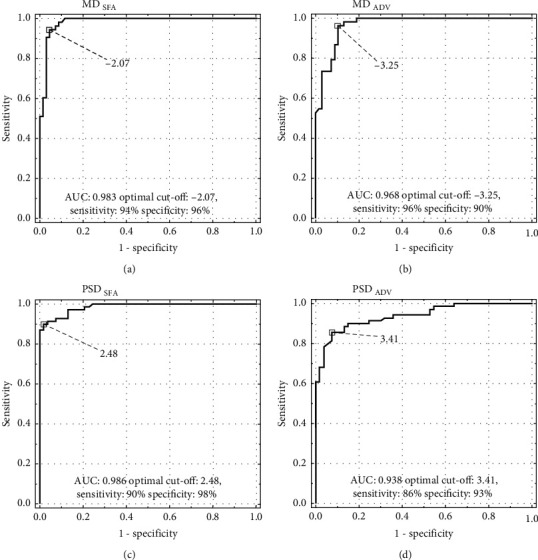
Receiver operating characteristic curves for MD (a, b) and PSD (c, d).

Analysis of ROC curve built from MD index shows bigger Area Under Curve (AUC) in SFA than in ADV (0.983 vs.0.968) (Figures 9(a) and 9(b)). The optimal cutoff point for SFA and ADV was −2.07 (sensitivity 94%, specificity 96%) and −3.25 (sensitivity 96%, specificity 90%), respectively. A larger difference can be seen when comparing the AUC in ROC of SFA and ADV built from the PSD index (0.986 vs. 0.938) (Figures 9(c) and 9(d). The optimal cutoff point for SFA and ADV was 2.48 (sensitivity 90%, specificity 98%) and 3.41 (sensitivity 86%, specificity 94%), respectively.

### 2.7. VFI and VQi

**Figure 10:**
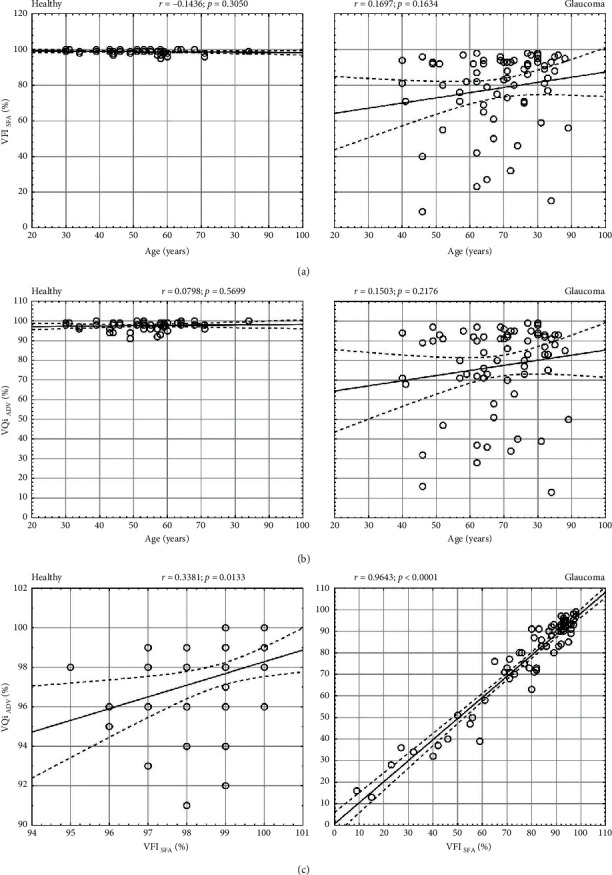
Visual field (VFI) and visual quality (VQi) indices analysis in the healthy and glaucoma group. (a, b) Correlation of VFI (for SFA) and VQi (for ADV) with patient age; (c) correlation between VQi and VFI.

There was a statistically significant difference in the mean values of VFI and VQi in the healthy group (*p* < 0.0001) but no difference in the glaucoma group (*p*=0.1036) (Table 1). The VQi was, on average, −1.26 ± 2.05% (VFi-VQi) lower than VFI in the healthy group and −1.16 ± 5.84% (VFI-VQi) lower than VFI in the glaucoma group. The difference was not significant (*p*=0.9009). There was no correlation between age and VFI (*r* = −0.14, *p*=0.3050) or age and VQi (*r* = 0.08, *p*=0.5699) in the healthy group. Similarly, there was no correlation between age and VFI (*r* = 0.17, *p*=0.1634) or age and VQi (r=0.15, *p*=0.2176) in the glaucoma group (Figures 10(a) and 10(b)). There was a very strong correlation between VFI and VQi in the glaucoma group (*r* = 0.96, *p* < 0.0001) but only a weak correlation in the healthy group (*r* = 0.34, *p*=0.0133) (Figure 10(c)).

### 2.8. GHT Sensitivity/Specificity

In the group of 53 healthy eyes, 37 SFA and 39 ADV assessments yielded a normal GHT result. Eleven SFA and only 2 ADV assessments were classified as a borderline GHT. The false-positive result, that is, GHT outside normal limits, was assigned to 5 SFA and 12 ADV results. When specificity was evaluated based on the normal and borderline GHT result considered a healthy outcome, the specificity of the SFA and ADV strategies was 90.57% and 77.36%, respectively. However, with the exclusion of the borderline GHT result as a healthy outcome, this value dropped to 69.81% and 73.58%, respectively. In the group of 69 glaucomatous eyes, 2 SFA and 3 ADV assessments yielded a false-negative result (that is, a normal GHT result). Three SFA and none of ADV assessments were classified as GHT borderline. The outside normal limits GHT was assigned to 64 SFA and 64 ADV results. When sensitivity was evaluated based on the outside normal limits GHT result as indicative of glaucoma, the sensitivity of the SFA and ADV strategies was 92.75% and 92.75%, respectively. However, with the inclusion of the borderline GHT result as indicative of glaucoma, the sensitivity of the SFA improved to 97.10% and the sensitivity of the ADV remained unchanged at 92.75%.

### 2.9. Sensitivity/Specificity of Anderson and Patella Criteria

In the group of 53 healthy eyes, 36 SFA and 37 ADV assessments met none of the 2 A&P criteria. There were 8 SFA and 6 ADV assessments, which met a single A&P criterion. The false-positive result (2 out of 2 A&P criteria met) was found in 9 SFA and 10 ADV assessments. When specificity was evaluated based on 0/2 A&P or 1/2 A&P criteria met considered a healthy outcome, the specificity of the SFA and ADV strategies was 83.02% and 81.13%, respectively. However, with the exclusion of 1/2 A&P criterion met as a healthy outcome, this value dropped to 67.92% and 69.81%, respectively. In the group of 69 glaucomatous eyes, 1 SFA and 4 ADV assessments yielded the false negative result (none of A&P criteria met). There were only 2 SFA and 9 ADV assessments which met a single A&P criterion. There were 66 SFA and 56 ADV assessments which met both A&P. When sensitivity was evaluated based on all A&P criteria met as indicative of glaucoma, the sensitivity of the SFA and ADV was 95.65% and 81.16%, respectively. However, with the inclusion of only one A&P criterion met as indicative of glaucoma, this value increased to 98.55% and 94.20%, respectively. All three A&P criteria, including the PSD probability <5%, were met in the SFA strategy by 64 patients from the glaucoma group. Using all 3 A&P criteria as indicative of glaucoma would increase specificity of SFA to 88.68% whist decreasing its sensitivity to 92.75%

## 3. Discussion

Both HFA II 750i and Optopol PTS 2000 offer gold standard parameters of visual field testing conditions. The equality in technical specification implies that the results obtained with one perimeter could be compared to those from another. Hence, one can assume that the differences demonstrated in this study solely originate from algorithm differences in SFA and ADV testing strategies.

The main goal of the SFA and ADV strategies is to minimize test duration while maintaining clinical capabilities of threshold testing. The previous studies showed that SFA can reduce test duration by 66% compared to the full threshold [27]. In this study, we found that test duration can be even shorter with the PTS 2000 ADV strategy. Compared to the SFA, the ADV test duration was reduced by 5% in healthy subjects and by 18% in patients with glaucoma. The ADV strategy also offers reduced variability of test duration, which leads to the conclusion that, with the ADV strategy, test duration is less affected by the patient's condition. The 18% shorter test duration for patients with glaucoma is a significant improvement, which reduces the fatigue effect [8–10]. It should be noted that there are other test strategies, which offer even greater test time reduction. The TOP strategy, whereby each test location is tested only once, enables test time shortening by 35% (in mixed healthy and glaucomatous subjects) whilst keeping the low variability (SD = 0.34) [28]. The reduced test duration comes at the expense of its accuracy. The TOP strategy tends to smooth the appearance of the visual fields underestimating localized defects [28]. The shortening of test time can also be achieved by using past examination data in order to adjust the starting levels of thresholding. This has been utilized in the ZATA strategy alongside the Bayesian method known from SITA [19]. However, the extra starting level adjustment based on historic data can only speed up testing of just 4 points compared to SITA or ADV strategies. Both SITA and ADV use a starting level calibration, and all newly tested points are tested from the calibrated level or from the level calculated based on adjacent points. Another specific characteristic of ZATA is the Bayesian stopping criterion which is variable and less strict for healthy visual field regions. This helps to reduce the test time further compared to SITA. As ZATE uses statistical methods, we may expect it to underestimate the defects in comparison to methods based on classical bracketing like full threshold, Fastpac or ADV [29]. One of such methods based on classical bracketing is the GATE algorithm. The test time reduction is gained not only by adjusting the starting levels but also by varying the bracketing step in deep or absolute defect areas. Using a bigger step not only reduces the accuracy but also minimises the number of exposures and test duration [18]. The same approach was also adopted in the Dynamic strategy [11]. A bigger step size is justified by a flatter slope of the frequency-of-seeing curve in areas of low visual sensitivity. The step varies between 2 and 10 dB depending on the actual defect. The 10 dB step size undoubtedly reduces testing accuracy in visual field defect areas. Unlike the GATE and Dynamic, the ADV strategy maintains the same precision throughout the entire sensitivity range.

A comparison of MS values obtained with SFA and ADV shows that, on average, the values obtained with the SFA are higher than those obtained with the ADV. This difference can be explained by the fact that the SFA strategy sets the final threshold at intensity values related to the peak of the probability distribution function. The peak of the function denotes intensity value with the largest probability to be a real threshold [16]. In contrast, the ADV strategy uses the last seen intensity as the final threshold. The phenomenon of increased MS obtained with the SITA strategies compared to the full threshold tests has been reported multiple times in previous studies [27, 29–32]. The differences in MS between the SFA and full threshold tests in these studies ranged from 1.3 dB in healthy subjects to 2.18 dB in patients with glaucoma [27, 29]. Therefore, based on the differences in MS values obtained with the SFA and ADV strategies, we are inclined to believe that the ADV results are closer to the full threshold results rather than to those of the SFA.

There was a very strong correlation (*r* = 0.95, *p* < 0.0001, *S* = 1.98 dB) between the MS values in ADV and SFA in patients with glaucoma. The MS is mainly affected by the testing algorithm and test parameters, such as stimulus intensity, background illumination, and stimulus size. Thus, if the parameters are matched and the testing algorithms are similar, we can expect to have a linear relation between the MS indices obtained with the two devices. This correlation was weaker (*r* = 0.77, *p* < 0.0001, *S* = 0.91 dB) in healthy subjects than in patients with glaucoma, in whom a higher variability of MS values assessed with the ADV than with the SFA was observed. This can be explained by the properties of the SITA algorithm which offers a lower variability of results for sensitivities above 20 dB than the full threshold, which is a noninteractive thresholding algorithm [30]. On the other hand, the healthy group, where the expected scatterplot has a high concentration of points around the normal value, cannot yield a very high correlation coefficient. In such a case, a standard error of regression (S) can be used to determine the association between two methods using an absolute measure. The value of *S* = 0.91 dB was calculated for the healthy group.

The RMS difference of threshold values between SFA and ADV was lower in the healthy group than in the glaucoma group, due to higher test-retest variability of results with deficits usually seen in glaucoma patients [27, 33]. The point locations plotted against RMS differences between the SFA and ADV on cumulative graphs in this study show that differences in healthy subjects ranged from 1.82 dB to 5.55 dB. The map with RMS values printed in test field locations reveals that the RMS value in healthy subjects increased with the eccentricity of the tested location. This can, again, lead to the conclusion that threshold variability in ADV strategy depends on the sensitivity threshold level, which normally decreases with eccentricity. In patients with glaucoma, however, the defect depth plays greater role than the usual sensitivity drop and, therefore, such correlation cannot be observed.

The PSD index describes an irregularity of visual field sensitivities. In our study, mean PSD values obtained with the ADV strategy in healthy subjects were slightly higher than those obtained with the SFA strategy. This can be explained by the higher variability of threshold values around the normal intensity in the ADV strategy than in the SFA. In the latter, one exposure is sufficient for some points to get the final threshold estimate. If the exposed brightness is close to the expected threshold estimate and the error-related factor is acceptable, the final threshold is set at that threshold estimate. As a result, the values close to the age norm are “snapped” to actual normative values, which reduces variability and irregularity. With the ADV strategy, on the other hand, the values are displayed, which the patient was actually exposed to and confirmed. Thus, there is no correlation between PSD assessed with ADV and SFA in the healthy group (*r* = 0.13, *p*=0.3424). However, in the glaucoma group, this effect is diminished. The SFA “snapping” is not that significant here, as the threshold values are already outside the normal range and the estimates are shifted towards lower intensities. In the glaucoma group, the mean difference in PSD indices between SFA and ADV is 0.53 dB. There is a very strong correlation between these values, confirmed by a high correlation coefficient (*r* = 0.86, *p* < 0.0001) and the trend line with a slope close to 1.

One of the latest global indices introduced in visual field analysis in HFA devices is VFI [34]. It is intended to present glaucoma progression rate whilst remaining less affected by cataract than the MD index. In the PTS 2000, the Visual Quality Index (VQi) plays exactly the same role. This parameter was introduced in order to quickly provide information on visual field defect progression with one percentage value in a readable format. A simplified description will, therefore, assume that the closer the VFI and VQi value is to 100%, the greater the patient's visual efficiency. The lower the value, the greater the impact of defects on the quality of vision. Our study showed a very high correlation between the VFI and VQi in the glaucoma group. In the healthy group, the VFI values were better concentrated around the mean, whilst the VQi spreads from 96% to 100%. This reconfirms a higher variability of ADV results in comparison to the SFA strategy within the normal range of sensitivities.

The sensitivity and specificity assessment, based on the GHT only, was conducted two-fold. In the first instance, a result was interpreted as indicative of glaucoma, if the GHT was outside normal limits. All borderline and normal results were assigned to the healthy group. Assessed based on a GHT outside normal limits result, the sensitivity and specificity of SFA were similar to those reported in previous studies [28]. In the second instance, a result was interpreted as indicative of glaucoma, if the GHT was outside normal limits or borderline. Using this criterion, the sensitivity of SFA increased (97.1%) and the specificity decreased to the level reported in previous studies [35, 36]. When the GHT result outside normal limits is considered the only criterion for abnormality, both SFA and ADV strategies have a high sensitivity but the specificity of the ADV is lower than that of the SFA by about 13%. This is due to the higher variability of results obtained in the healthy group, with some test points falling outside the normal range which results in the GHT outside its normal limits. However, when the borderline GHT result is also considered abnormal, the specificity of SFA decreases significantly and it is even lower than that of the ADV. In this case, some results from the healthy group which are borderline at the SFA are just assigned to outside normal limits by the ADV.

The criteria proposed by A&P are widely used to classify visual fields as normal or glaucomatous. [5] The sensitivity assessment which considers results with 2/2 A&P criteria fulfilled to be glaucomatous shows that the SFA is a more sensitive testing algorithm (95.65%) than the ADV (81.16%) whilst both offer comparable specificity (83.02% vs. 81.13% for the SFA and ADV, respectively). The sensitivity assessment which considers results with, at least, 1/2 A&P criteria fulfilled to be glaucomatous yielded increased values for both SFA and ADV, with SFA sensitivity being higher (98.55%) than that of the ADV (94.20%). The specificity was very similar for SFA (67.92%) and ADV (69.81%). Low specificity values may suggest a high number of artefacts interfering with result accuracy in healthy subjects in both strategies, as a result of which many normal results were classified as glaucomatous. This tendency can be acceptable in rapid tests which do not take longer than screening tests. On the other hand, high sensitivity with low specificity increases the practice load and can lead to misdiagnoses and unnecessary long-term treatment with substantial adverse health outcomes and economic burden.

Due to the differences between algorithms, the ADV strategy produces different results to those of the SFA. Since ADV is more sensitive to patient invalid responses in the normal range of sensitivities, the visual field can be classified as outside normal limits. At the same time, the SFA strategy adjusts the patient result to age norm and, therefore, keeps healthy patient visual field closer to it. The ADV strategy in its core is more similar to the HFA full threshold test as its sensitivity results have values that have been tested and have evoked a response. The SFA due to its roots in statistics and probability theory gives results which are not always measured but are the most probable. This makes it less prone to small fluctuations and patient errors and gives the SFA a better measure of specificity based on the GHT criterion. The problem of high variability of sensitivity results due to patient response inconsistency has been known and addressed in the past [1, 3]. One of the latest testing approaches with promising results is the SPARK strategy [20]. The algorithm provides averaged results derived from the intermediate results obtained throughout the four testing stages. The method is based on statistical analysis of the relationship between sensitivities of the adjacent retinal points. The obtained results prove to reduce the intratest variability and test-retest reproducibility.

The differences between the two strategies lose their importance when evaluating glaucomatous fields because the normal variability of evaluated sensitivities within the glaucoma-affected regions is much higher than in healthy regions. The differences between strategies in glaucomatous fields are then less noticeable and seem negligible. This can be confirmed by the high sensitivity of both strategies when using both GHT and A&P criteria. Therefore, in light of that mentioned above, the further study could aim to directly compare SFA and ADV with full threshold strategy. This will help to determine which strategy provides closer estimations of the full threshold sensitivities. Another aspect that could be covered in further studies is the results reproducibility. The test with repeated examinations of the same patients may give the answer whether the ADV strategy is actually prone to fluctuations more than statistically supported SFA. Finally, it would be also beneficial to compare the ADV to the SFR. The latter strategy was not available yet when the study was carried out.

Both SFA and ADV are fast strategies which enable successful identification of glaucomatous defects. Our study has demonstrated that both algorithms offer the same high sensitivity of 92.5% which has not been compromised by test duration. Both strategies enabled effective identification of glaucomatous defect within 5 minutes with the ADV, where the assessment was shorter by 1 minute, being the fastest strategy in the study. However, the ADV strategy has one shortcoming compared to the SFA. Its results are not based on statistical data; therefore, the test-retest variability cannot be reduced in healthy patients, as it is carried out with the SFA. As a result, the ADV algorithm offers lower specificity when evaluated using the specificity criteria designed for SITA algorithms and, thus, requires more retests to make certain diagnosis.

## Figures and Tables

**Table 1:** Visual field indices values for the SFA and ADV strategies in healthy and glaucomatous eyes.

	Healthy subjects (*n* = 53)			Glaucoma patients (*n* = 69)		
	SFA	ADV	*p* value	SFA	ADV	*p* value
Test duration (s)	178 ± 17	169 ± 16	*p*=0.0053	261 ± 57	213 ± 35	*p* < 0.0001
MS (dB)	30.28 ± 1.43	29.22 ± 1.78	*p* < 0.0001	20.94 ± 6.36	19.93 ± 5.98	*p* < 0.0001
MD (dB)	−0.34 ± 1.12	−1.08 ± 1.46	*p*=0.0021	−8.83 ± 6.93	−9.68 ± 6.15	*p* < 0.0001
PSD (dB)	1.52 ± 0.38	2.38 ± 0.64	*p* < 0.0001	6.76 ± 3.76	6.23 ± 3.03	*p*=0.0039
VFI (VQi) (%)	98.9 ± 1.2	97.6 ± 2.1	*p* < 0.0001	78.3 ± 21.7	77.1 ± 22.0	*p*=0.1036

## Data Availability

Data used to support the findings of this study are available from the corresponding author on request.
